# Prediabetes and risk of heart failure: the link grows stronger

**DOI:** 10.1186/s12933-021-01302-w

**Published:** 2021-05-24

**Authors:** Jian-di Wu, Dong-liang Liang, Yue Xie

**Affiliations:** Department of Cardiology, Affiliated Foshan Hospital, Southern Medical University, The Second People’s Hospital of Foshan), Weiguo Road, Chancheng District, Foshan, 528000 PR China

**Keywords:** Prediabetes, Heart failure, Risk, Morbidity

## Abstract

In a recently published paper in *Cardiovascular Diabetology*, Sinha et al. (Association of fasting glucose with lifetime risk of incident heart failure: the Lifetime Risk Pooling Project. Cardiovasc Diabetol. 2021;20(1):66) reported that prediabetes (defined as a fasting plasma glucose concentration of 100–125 mg/dL) was associated with a higher lifetime risk of heart failure in middle-aged White adults and Black women, with the association attenuating in older Black women. This study provides important evidence that the risk of heart failure is increased in people with a fasting plasma glucose concentration as low as 100 mg/dL, supporting the definition of prediabetes according to the American Diabetes Association guideline. The study also strongly supports the notion that prediabetes should be regarded not only as a high-risk state for the development of diabetes but also as a risk factor for cardiovascular morbidity.

Prediabetes, also termed “intermediate hyperglycemia,” is an intermediate metabolic state between type 2 diabetes mellitus (T2DM) and normoglycemia and includes patients with impaired fasting glucose (IFG) and impaired glucose tolerance (IGT) [1, 2]. IFG is defined as an elevated fasting glucose concentration, and IGT is defined as a high 2-h plasma glucose concentration during an oral glucose tolerance test that does not reach the diagnostic cut-off point for diabetes. Hemoglobin A1c (HbA1c)-based definitions of prediabetes have also recently been advocated. Although individuals in all three of these categories have been shown to have an increased risk of progression to T2DM, each condition has a different pathophysiology that does not completely overlap with the others [3]. Furthermore, the cut-off points for diagnosing prediabetes using the fasting plasma glucose (FPG) and HbA1c concentrations remain controversial. Great debate has surrounded the lower cut-off point proposed by the American Diabetes Association (ADA) guideline, which recommends using an FPG concentration of 100 to 125 mg/dL or HbA1c concentration of 5.7–6.4% to define prediabetes [4]. The counterargument is that the definition of prediabetes proposed by the ADA guideline significantly increases the prevalence of blood glucose dysregulation with no clear association with diabetic complications. Although meta-analyses of observational studies have provided solid evidence that prediabetes is associated with an increased risk of cardiovascular disease and mortality, controversy remains [5, 6].

In a recently published paper in *Cardiovascular Diabetology*, Sinha et al. [7] analyzed 40,117 participants from 6 population-based cohorts in the United States. They found that prediabetes (defined as an FPG concentration of 100–125 mg/dL) was associated with a higher lifetime risk of heart failure in middle-aged White adults and Black women, whereas the association was attenuated in older Black women [7]. This study strongly supports the notion that prediabetes should be regarded not only as a high-risk state for the development of diabetes but also as a risk factor for cardiovascular morbidity. Interestingly, at almost the same time as publication of the study by Sinha et al. [7], Cai et al. [8] published a meta-analysis showing that the risk of heart failure was higher in individuals with prediabetes than in those with normoglycemia. That meta-analysis included 15 observational studies comprising nearly 10 million individuals with a median follow-up duration of 8.0 years for analysis [8]. The results showed that prediabetes, defined as IFG, IGT, or an elevated HbA1c concentration, was associated with an increased risk of heart failure after adjusting for multiple cardiovascular risk factors. Importantly, the increased risk was observed when the FPG concentration was as low as 100–125 mg/dL or the HbA1c was 5.7% to 6.4%, according to the ADA’s proposed definition of prediabetes. Furthermore, in adults with diabetes and prediabetes, a biomarker score can stratify the risk of heart failure and inform the allocation of heart failure prevention therapies [9]. Together, these studies strongly support use of the term “prediabetes” from the viewpoint of preventing a common diabetic complication: heart failure.

Notably, individuals with prediabetes are more likely to progress to T2DM. Therefore, the risk of heart failure may be confounded by progression to T2DM. Unfortunately, these studies did not adjust for such confounders. In future studies, a series of blood glucose measurements is needed in individuals with prediabetes to determine the blood glucose trajectory and the association of diabetic complications, including heart failure. Furthermore, it is known that lifestyle interventions in people with prediabetes can reduce the risk of cardiovascular events and all-cause mortality [10]. It is also important to determine the risk of heart failure in individuals with prediabetes who are undergoing lifestyle interventions.

## Data Availability

Not applicable.

## Abbreviations

ADA: American Diabetes Association
FPG: Fasting plasma glucose
HbA1c: Hemoglobin A1c
IFG: Impaired fasting glucose
IGT: Impaired glucose tolerance
T2DM: Type 2 diabetes mellitus
